# Fenofibrate Reduces Mortality and Precludes Neurological Deficits in Survivors in Murine Model of Japanese Encephalitis Viral Infection

**DOI:** 10.1371/journal.pone.0035427

**Published:** 2012-04-13

**Authors:** Neha Sehgal, Kanhaiya Lal Kumawat, Anirban Basu, Vijayalakshmi Ravindranath

**Affiliations:** Division of Molecular and Cellular Neuroscience, National Brain Research Centre, Nainwal Mode, Manesar, Haryana, India; Cincinnati Childrens Hospital Medical Center, United States of America

## Abstract

**Background:**

Japanese encephalitis (JE), the most common form of viral encephalitis occurs periodically in endemic areas leading to high mortality and neurological deficits in survivors. It is caused by a flavivirus, Japanese encephalitis virus (JEV), which is transmitted to humans through mosquitoes. No effective cure exists for reducing mortality and morbidity caused by JEV infection, which is primarily due to excessive inflammatory response. Fenofibrate, a peroxisome proliferator-activated receptor-α (PPARα) agonist is known to resolve inflammation by repressing nuclear factor-κB (NF-κB) and enhancing transcription of anti-oxidant and anti-inflammatory genes. In addition, fenofibrate also up-regulates a class of proteins, cytochrome P4504Fs (Cyp4fs), which are involved in detoxification of the potent pro-inflammatory eicosanoid, leukotriene B_4_ (LTB_4_) to 20-hydroxy LTB_4_.

**Methodology/Principal Findings:**

The neuroprotective effect of fenofibrate was examined using in vitro (BV-2 microglial cell line) and in vivo (BALB/c mice) models of JEV infection. Mice were treated with fenofibrate for 2 or 4 days prior to JEV exposure. Pretreatment with fenofibrate for 4 but not 2 days reduced mortality by 80% and brain LTB_4_ levels decreased concomitantly with the induction of Cyp4f15 and 4f18, which catalyze detoxification of LTB_4_ through hydroxylation. Expression of cytokines and chemokine decreased significantly as did microglial activation and replication of the JEV virus.

**Conclusions/Significance:**

Fenofibrate confers neuroprotection against Japanese encephalitis, in vivo, in mouse model of JEV infection. Thus, fenofibrate, a PPARα agonist that is commonly used as a hypolipidemic drug could potentially be used for prophylaxis during JE epidemics to reduce mortality and morbidity.

## Introduction

JEV, a flavivirus causes acute encephalopathy in children [1], [2], [3]. A large geographical area including India, China, Japan and most of South East Asia are afflicted by Japanese encephalopathy (JE). The incidence of JE in endemic areas of Asia is nearly 50,000 cases per year. High mortality, representing over 20% has been reported. The clinical manifestations of infection include fever, headache, vomiting, signs of meningeal irritation, and altered consciousness [4]. JEV infection thus, targets the CNS leading to high mortality. In survivors, the morbidity is high due to lingering neurological and/or psychiatric deficits in a large proportion of patients [5]. Current therapeutic interventions for JE are symptomatic and no cure is available.

The principle cells, which respond to JEV infection are the microglia in the CNS. Microglia are known to be involved in the immune surveillance of the brain. They are the immune effector cells and undergo extensive morphological changes from resting to activated state following JEV challenge. Morphological changes are accompanied by extensive proliferation and chemotaxis resulting in release of mediators that trigger massive inflammatory response [6], [7]. Thus, JEV infection in CNS is characterized by extensive recruitment of microglia followed by release of pro-inflammatory molecules that are the prime mediators of neuropathological changes associated with JE [8].

Inflammatory response in JE acts as a double-edged sword where it protects from initial damage and is involved in repair process leading to neuroprotection. However, extensive microglial activation acts as a driving force resulting in irreversible damage. The activated microglia release excessive amounts of cytokines (TNF-α, IL-6, IL-1β etc.), chemokines (MCP-1, MIPα etc.), eicosanoids including leukotrienes, particularly leukotriene B_4_ (LTB_4_) and prostaglandins [9], [10]. A complex interplay exists between eicosanoids including leukotrienes, prostaglandins produced from arachidonic acid on one hand and cytokines/chemokines on the other [11], [12]. Thus, limiting the extensive microgliosis accompanying JE could potentially halt the progression of events leading to mortality and morbidity caused by JEV infection.

Peroxisome proliferator-activated receptor-α (PPARα) is one of the members of nuclear receptor PPAR family and is a modulator of inflammation [13], [14]. Fenofibrate is an agonist of PPARα and leads to its activation promoting the expression of neuroprotective genes, which trigger both anti-oxidant and anti-inflammatory response [15], [16]. It increases the transcription of a number of genes such as Cu/Zn^2+^ superoxide dismutase (SOD), glutathione peroxidase (GPx), glutathione reductase (GR), glutathione-S-transferase (GST), catalase and interferes at the transcriptional level with nuclear factor-κB (NF-κB), signal transducers and activators of transcription (STATs), activator protein 1 (AP1), nuclear factor of activated T cells (NFAT) signaling pathways [17], [18], [19], [20]. PPARα activation by fenofibrate confers neuroprotection in various CNS disorders such as traumatic brain injury and Parkinson's disease [18], [20]. In addition, we have recently shown that fenofibrate also induces another family of anti-inflammatory genes, namely the Cyp4fs that are involved in the metabolism of potent pro-inflammatory eicosanoid, LTB_4_ to the non-toxic 20-hydroxy LTB_4_. The induction of Cyp4fs results in increased metabolism of the inflammatory prompt LTB_4_ and confers neuroprotection against LPS mediated damage [21]. Thus, induction of Cyp4fs by fenofibrate in brain plays an important role in regulating the inflammatory response. In the present study we examined the neuroprotective effect of fenofibrate using both in vitro and in vivo models of JEV infection and demonstrate the significant neuroprotection offered by fenofibrate through reduction of inflammation and viral replication.

## Methods and Materials

### Statement for animal ethics

Animal experiments were carried out on adult BALB/c mice, (4–6 weeks old, 16–20 g) procured from National Brain Research Centre (NBRC). All animal experiments were carried out according to institutional guidelines for the use and care of animals. Animal experiments were approved by the institutional animal ethical review board, named “Institutional Animal and Ethics Committee of National Brain Research Centre”. The animal experiment protocol approval number is NBRC/IAEC/2007/36. Handling of animals was done according to the guidelines of Committee for the Purpose of Control and Supervision of Experiments on Animals (CPCSEA), Ministry of Environment and Forests, Government of India. Animals had access to pelleted diet and water, *ad libitum*.

### Materials

Antibody to Iba-1 was procured from Wako Pure Chemical Industries Ltd. (Osaka, Japan). Antigen unmasking solution, conjugated secondary antibody, Vectastain-ABC Elite kit and Nova Red substrate kit were purchased from Vector laboratories (Burlingame, CA). Cell culture products and Lipofectamine™ were obtained from Gibco BRL (Invitrogen, Carlsbad, CA). TRI reagent and bromochloropropane (BCP) were purchased from Molecular Research Centre (Cincinnati, OH, USA). Single-stranded cDNA synthesis kit and SYBR Green supermix were purchased from Applied Biosystems (Foster city, CA 94404 USA). Fenofibrate was obtained from Sigma Chemical Company (St. Louis, MO). Cytokine bead array (CBA) mouse inflammation kit was obtained from BD Biosciences (USA). The Assay designs™ leukotriene B_4_ enzyme immunoassay kit was procured from Stressgen (Michigan; USA). Microslides (superfrost plus) were obtained from VWR International (USA). All other chemicals or reagents were of analytical grade and were obtained from Sigma Aldrich, Merck or Qualigens (India).

### Treatment schedule

BALB/c mice were injected with the JEV strain, GP78 (3–10^5^ pfu in phosphate buffered saline; intravenously) as described previously [7]. Control animals received phosphate buffered saline (PBS). For examining the neuroprotective effect of fenofibrate, animals were divided into four groups. Two groups received vehicle for 2 or 4 days followed by intravenous injection of PBS or JEV on the fourth day, 1 hr after the vehicle. Treatment with vehicle was continued for another 9 days post-injection in both groups. The remaining two groups received fenofibrate (100 mg/kg body weight; subcutaneous) for 2 or 4 days followed by intravenous injection of JEV or PBS, 1 hr after fenofibrate on the fourth day. The fenofibrate treatment in these two groups was continued for another 9 days post-injection. For mortality study, animals were divided into four groups with 15 animals each and the survival of animals in each group was recorded up to 10 days post-infection. For biochemical analysis, animals were divided into four groups with 6 animals each, sacrificed on the sixth day post-injection and the cortex was dissected out. All efforts were made to minimize suffering, to reduce the number of animals used, and to utilize alternatives to in vivo techniques where available.

### Behavioral Scoring

Animals infected with virus developed mild symptoms after 4 days, which worsened progressively and most animals died on 9^th^ day post-infection. Symptoms of JE included pilo-erection, restriction of movements, poor pain response, stooping posture, whole body tremor, body stiffening, and hind limb paralysis. The severity of infection was analyzed by scoring the animals for the progression of symptoms. The scoring done was as follows: **Score 0** = No restriction in movement, no pilo-erection, no body stiffening and no hind limb paralysis; **Score 1** = No restriction in movement, no body stiffening, no hind limb paralysis but *pilo-erection* and *slowless in movement* were observed; **Score 2** = No restriction in movement, no body stiffening, no hind limb paralysis but *pilo-erection*, *slowless in movement*, *slight hind limb extension*, *and stooping posture* were observed; **Score 3** = *Restriction in movement, pilo-erection, mild body stiffening, slight body jerks, slight hind limb extension but* no hind limb paralysis were observed; **Score 4** = *Restriction in movement, pilo-erection, body stiffening, hind limb paralysis, occasional tremor*; **Score 5** = *Restriction in movement, pilo-erection, body stiffening, hind limb paralysis, tremor, which were followed by the animals succumbing to death*. For recording limb clasping reflex, mice were lifted by tail and the ability of the animals to extend the hind limbs was recorded. All other neurological parameters were recorded visually and the total score was calculated on the basis of the appearance of the symptoms.

### Immunohistochemistry

Animals were perfused transcardially with buffered paraformaldehyde (4% w/v), 24 hr after the last dose, the brain was dissected out and processed for immunohistochemistry. Brains were sectioned on a cryostat and immunohistochemistry was done as described earlier [21]. Serial coronal sections (30 µm thick) were cut throughout the entire cortex and hippocampus. Cryosections were mounted on microslides and air-dried. The sections were washed with tris-buffered saline (TBS) and quenched with 3% hydrogen peroxide (v/v) for 20 min. Sections were then washed with TBS and subjected to heat induced epitope retrieval using antigen unmasking solution as per the manufacturer's protocol. Sections were washed and blocked with TBS containing 3% (v/v) normal goat serum, 2% BSA (w/v) and 0.1% Triton X (v/v) for 2 hr at room temperature. The cryosections were then washed and incubated overnight with the primary antibody to Iba-1 at 4°C. The sections were washed and incubated serially with biotinylated anti-rabbit antibody and avidin-biotin conjugated horseradish peroxidase while ensuring that the sections were washed well between incubations. Immunostaining was visualized following development with the peroxidase substrate, Nova Red. Sections were dehydrated by exposure to increasing gradient of alcohol and finally in xylene and cover-slipped using DPX (a mixture of **d**istyrene, a **p**lasticizer, and **x**ylene) as the mounting medium. Images were captured using Leica DMRXA2 microscope. Non-immune IgG was used in place of immune serum as negative control for immunohistochemistry. For quantitation of microglia, sections were immunostained with Iba-1 antibody. Every 4th section and a total of 20 sections were used for stereological assessment from each animal. Stereological analysis was done by determining number of Iba1-activated microglia in the cortex per square millimeter using IM50 software (Leica).

### Processing of tissue for biochemical analysis

The animals were decapitated and the cortex was dissected out. Cortex was homogenized in potassium phosphate buffer containing 0.25 M sucrose and protease inhibitor cocktail. The homogenate was centrifuged at 100,000× g for 1 hr to obtain the cytosol. Protein concentration was estimated by dye binding method [22]. Cytosol was used for cytokine profiling, estimation of leukotriene B_4_ (LTB_4_) levels and plaque assay to quantify viral replication.

### Cell-culture experiments

BV-2, murine microglial cells, which exhibit both phenotypic and functional properties of reactive microglial cells were grown in DMEM supplemented with 10% FBS, streptomycin and penicillin. BV-2 cells were treated with fenofibrate (40 µg/ml) 1 hr prior to exposure with JE virus. Cells were then exposed to live virus (multiplicity of infection = 5) for 1 hr [6]. Unbound virus was removed by gentle washing with PBS and fenofibrate treatment was continued for 24 hr. The supernatant was used for quantifying cytokines and chemokine using cytokine bead array, LTB_4_ assay by ELISA and plaque assay for viral replication.

### Estimation of cytokines, chemokine and LTB_4_

Cytosol prepared from the mouse brain cortex was used for quantitation of cytokines and chemokine levels using cytokine bead array (CBA, mouse inflammation kit). The assay was performed according to the manufacturer's instructions and analyzed on the FACS Calibur machine (Becton Dickinson). Following acquisition of data, BD™ CBA Analysis Software that allows the calculation of cytokine concentrations in unknown samples was used for quantitation [23]. LTB_4_ levels were quantitated in mouse brain cytosol and cell culture supernatant using the Assay Designs™ Leukotriene B_4_ enzyme immunoassay kit according to manufacturer's instructions.

### RNA isolation and quantitative real time-PCR

Total RNA was isolated from BV-2 cells or mouse brain cortex of mice using TRI reagent [24]. Random hexamers were used for cDNA synthesis using High-Capacity cDNA Reverse Transcription Kit. Real-time PCR was performed on ABI 7500 sequence detection system using Power SYBR Green PCR Master Mix according to the manufacturer's instructions. All reactions were carried out in triplicate and negative control without the template was also included. 18S rRNA was used as an internal control for normalization. Data was analyzed using the comparative threshold cycle (ΔΔCt) method. The RNA levels of each gene were normalized to the respective 18S levels. Following normalization, relative mRNA values were normalized using one of the genes or treatment conditions. Dissociation curves were generated to check for the specificity of primers. The relative efficiency of each PCR reaction was calculated from the respective standard curve. The sequence of the primers used for quantitative real time-PCR, amplicon size, slope and relative efficiencies of each reaction is mentioned in Table 1.

**Table 1:** LIST OF PRIMERS USED FOR QRT-PCR.

GENE	PRIMER SEQUENCE	SIZE	SLOPE EFFICIENCY
Cyp4f13	Forward 5′ CATCTTGGATTCTAGCCCGAA 3′Reverse 5′ GAAGCAACGAAGGCGACTG 3′	74 bp	−3.23104%
Cyp4f14	Forward 5′ ACTGGCTTATGGGTCACGTG 3′Reverse 5′ ACCCACCAAACGAGTCAATTC 3′	74 bp	−3.31101%
Cyp4f15	Forward 5′ CATGACATGGCTGGGTCCTA 3′Reverse 5′ GAGGCATTGAGAACAGATCGA 3′	78 bp	−3.3499%
Cyp4f16	Forward 5′ CCTTGCCTGGACTTACTCATT 3′Reverse 5′ GTAACCAGCTGCATGCCTTC 3′	132 bp	−3.36102%
Cyp4f18	Forward 5′ AGAGCCTGGTGCGAACCTT 3′Reverse 5′ TGGAATATGCGGATGACTGG 3′	73 bp	−3.32100%
TNF-α	Forward 5′ ATGGCCCAGACCCTCACACTCA 3′Reverse 5′ ACAACCCATCGGCTGGCACCA 3′	184 bp	−3.22104%
IL-6	Forward 5′ ACCTGCTGGTGTGTGACGTTC 3′Reverse 5′ GTCGTTGCTTGGTTCTCCTTGTAC 3′	179 bp	−3.27102%
MCP-1	Forward 5′ GCTGTAGTTTTTGTCACCAAGCTCAA 3′Reverse 5′ TGAAGACCTTAGGGCAGATGCAG 3′	150 bp	−3.33100%
IL-1β	Forward 5′ ACCTGCTGGTGTGTGACGTTC 3′Reverse 5′ GTCGTTGCTTGGTTCTCCTTGTAC 3′	179 bp	−3.28102%
GP-78	Forward 5′ TTGACAATCATGGCAAACGA 3′Reverse 5′ CCCAACTTGCGCTGAATAAT 3′	200 bp	−3.32100%

### Assay of virus replication

Virus titration was performed to confirm the presence or absence of live virus in the supernatant collected from BV-2 cells or cytosol prepared from mouse brain cortex. Plaque formation on monolayer of porcine stable kidney cells (PS) was monitored for quantifying the viral titre. PS cells were seeded in six well plates to form semi-confluent monolayer and then inoculated with 10 fold serial dilutions of cell culture supernatant or mouse brain cytosol. Serial dilutions were made in MEM containing 1% fetal calf serum and incubated for 1 hr at 37°C with occasional shaking. The inoculum was removed by aspiration and the monolayers were overlaid with MEM containing 4% fetal calf serum, 1% low-melting point agarose and a cocktail of antibiotic-antimycotic solution containing penicillin, streptomycin and amphotericin B. Plates were incubated at 37°C for 3–4 days until plaques became visible. The cells were fixed with 10% paraformaldehyde and plaques were quantified following staining with 0.1% crystal violet [25].

### Data analysis and Statistics

Statistical Analysis of the data was performed using Sigma Stat 3.5 software and Sigma Plot 10.0 for generating graphs. Significance was assessed using One-Way Analysis of Variance followed by post-hoc tests (Student-Newmann Keul's or Bonferroni) for multiple comparisons and Student's ‘*t*’ test for comparison between two groups. Values of p<0.05 were taken to be statistically significant. Data is represented as mean ± standard deviation.

## Results

### Fenofibrate abrogates the JEV mediated microglial activation and release of inflammatory mediators in BV-2 cells

**Figure 1:**
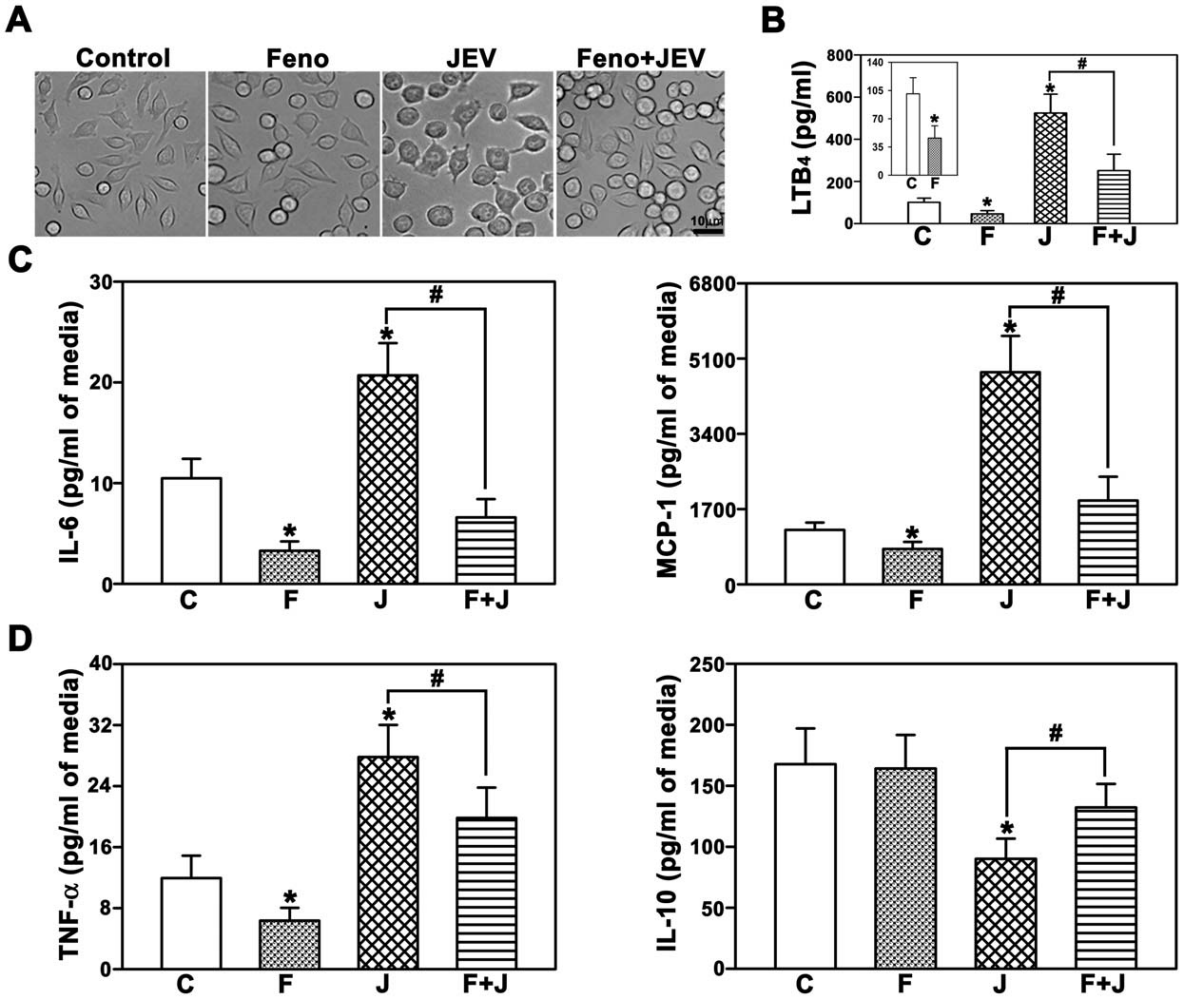
Inflammatory response to Japanese encephalitis virus (JEV), in vitro, is abrogated by fenofibrate. BV-2 cells were treated with vehicle (C), fenofibrate (Feno, F), JEV (J) or both fenofibrate and JEV (Feno+JEV, F+J). (A) Fenofibrate attenuates the morphological changes induced by JEV in BV-2 cells as seen in the phase contrast images. Treatment of BV-2 cells with fenofibrate markedly reduced the levels of LTB_4_ (B), IL-6, MCP-1 and TNF-α (C,D) released into the medium after JEV infection. Conversely, protective cytokine IL-10 production was increased in fenofibrate (D) pretreated cells compared to JEV alone (n = 6 per group, *and #p<0.05). Fenofibrate alone results in decreased levels of LTB_4_ (inset in B) and cytokines and chemokine (C,D).

In order to assess the role of Cyp4fs in attenuating the massive inflammatory response induced by JEV infection, we first used a cell culture approach. JEV infection of BV-2, the mouse derived microglial cells triggered massive inflammatory response as seen by the morphological changes (Figure 1A) and release of pro-inflammatory mediators, particularly LTB_4_ (Figure 1B), cytokines and chemokine (IL-6, MCP-1, TNF-α; Figures 1C and 1D) in the medium and significant reduction of the protective cytokine, IL-10 levels (Figure 1D). Pretreatment with fenofibrate, an inducer of Cyp4fs attenuated JEV mediated release of LTB_4_, cytokines and chemokine (IL-6, MCP-1, TNF-α; Figures 1B,1C and 1D). Interestingly, fenofibrate alone inhibited the release of constitutive LTB_4_ (inset in Figure 1B), and cytokines and chemokine (IL-6, MCP-1, TNF-α; Figures 1C and 1D).

### Induction of Cyp4fs by fenofibrate attenuates inflammatory response

**Figure 2:**
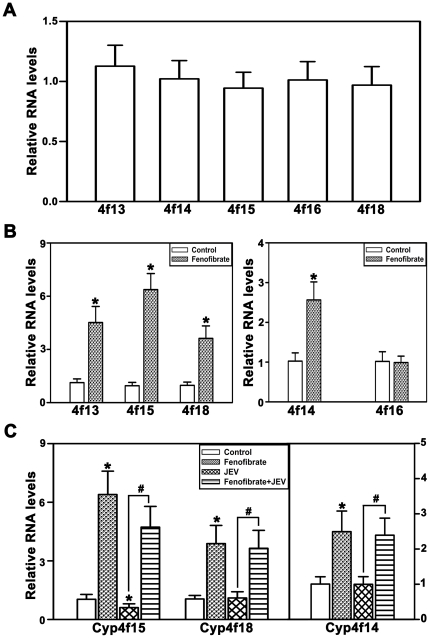
Induction of Cyp4fs by fenofibrate, in vitro, in BV-2 cells. (A) Constitutive expression of Cyp4fs in BV-2 cells. mRNA levels of all Cyp4fs are expressed relative to Cyp4f15. (B) Fenofibrate induced the expression of Cyp4f13, 14, 15 and 18, however the levels of Cyp4f16 were unaltered. (C) There was a significant decrease in the expression of Cyp4f15 following JEV infection. The levels of Cyp4f14 and 18 were unchanged. Pretreatment with fenofibrate however, attenuated JEV mediated inhibition of Cyp4f15. The mRNA levels in B, C are expressed relative to controls (n = 6 per group, *and #p<0.05).

In order to ascertain whether the protective effect conferred by fenofibrate is through the induction of Cyp4fs, we quantitated the levels of Cyp4fs in BV-2 cells. BV-2 constitutively expresses Cyp4f13, 4f14, 4f15, 4f16 and 4f18 (Figure 2A). Treatment with fenofibrate for 24 hr resulted in substantial induction of Cyp4f13, Cyp4f14, Cyp4f15 and Cyp4f18, while Cyp4f16 was unaltered (Figure 2B). Though all Cyp4fs are expressed in BV-2 cells we focused on Cyp4f14, Cyp4f15 and Cyp4f18 as they metabolize LTB_4_ to 20-hydroxy LTB_4_ efficiently while Cyp4f13 and 16 are poor metabolizers [21]. Expression of Cyp4f15 decreased substantially whereas of Cyp4f14 and Cyp4f18 were unchanged following JEV infection in BV-2 cells (Figure 2C). However, following fenofibrate pretreatment there was a significant increase in the levels of Cyp4f14, Cyp4f15 and Cyp4f18 compared to JEV alone (Figure 2C). Thus, fenofibrate may provide protection against JEV-mediated inflammatory response by inducing the expression of Cyp4fs particularly Cyp4f15 that was down-regulated during JEV infection so as to enable the efficient detoxification of LTB_4_.

### Fenofibrate confers protection against JEV infection, in vivo in mice

**Figure 3:**
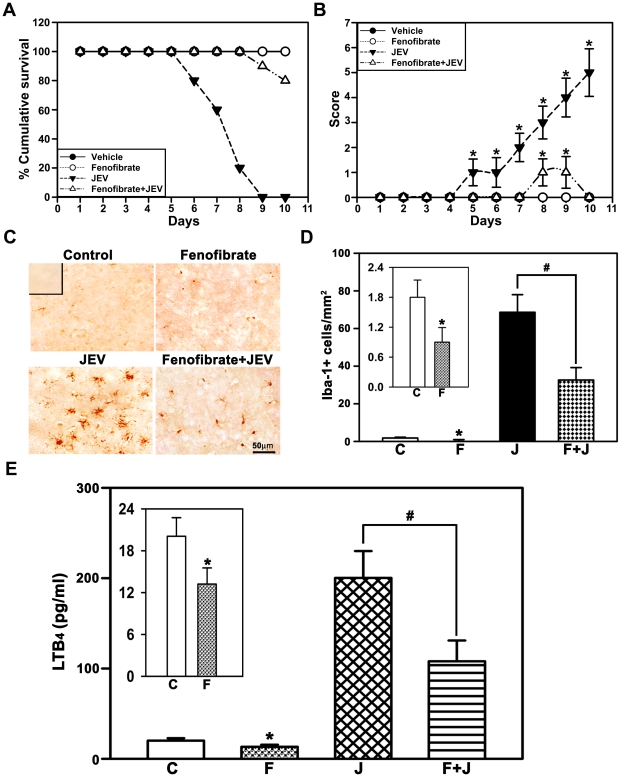
Fenofibrate protects against JEV infection, in vivo, in mice. (A) Fenofibrate markedly reduced the mortality in JEV infected animals (n = 15 per group). (B) Scores depicting the reversal of behavioral deficits following fenofibrate treatment compared to JEV infection alone (n = 15 per group, *p<0.05). (C,D). Stereological analysis using immunolabeling with antibody to Iba-1 showed significant decrease in number of activated microglia following pretreatment with fenofibrate compared to JEV alone. Inset depicts the negative control for immunohistochemical localization of Iba-1, which was performed using normal rabbit IgG. (E) Pretreatment with fenofibrate decreased the JEV mediated increase in LTB_4_ levels in the cortex. Data is represented as (C) vehicle control, (F) fenofibrate, (J) JEV, (F+J) fenofibrate+JEV treated mouse brain cortex (n = 6 per group, *and #p<0.05). Treatment with fenofibrate alone results in decreased number of activated microglia (inset in D) and levels of LTB_4_ (inset in E).

**Figure 4:**
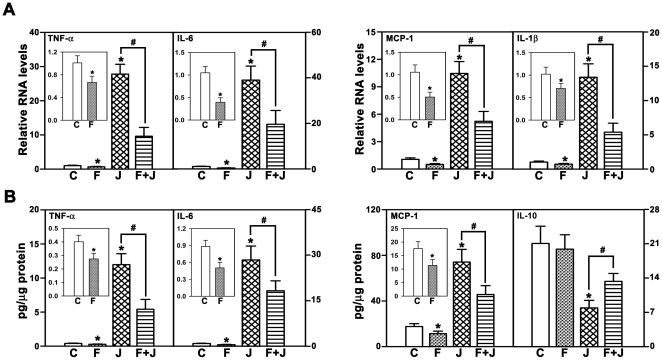
Fenofibrate attenuates the massive inflammatory response seen in JEV infection. The inflammatory response to JEV challenge was attenuated by fenofibrate as depicted by significant reduction in mRNA expression (A) and protein levels (B) of cytokines (TNF-α, IL-6, IL-1β) and chemokine (MCP-1) compared to JEV alone. Conversely, protective cytokine IL-10 (B) production was increased in fenofibrate treated mice compared to JEV alone. Insets in A,B show the decrease in cytokines and chemokine after fenofibrate treatment alone. Data is represented as (C) vehicle control, (F) fenofibrate, (J) JEV, (F+J) fenofibrate+JEV treated mouse brain cortex. mRNA levels in A,B for all treatments are expressed relative to controls (n = 6 per group, *and #p<0.05).

We then examined the effect of fenofibrate in vivo, using a murine model of JEV infection, which is characterized by neurological deficits potentially resulting from the massive inflammatory response. Fenofibrate pretreatment provided extraordinary protection against JEV induced mortality, wherein mortality was decreased by 80% (Figure 3A). The onset and progression of neurological symptoms evident by the behavioral scores, abnormal limb-clasping reflexes and behavioral deficits induced by JEV infection were not seen in fenofibrate treated mice (Figure 3B). Fenofibrate substantially reduced JEV-mediated massive inflammatory response seen as decrease in number of activated microglia (Figures 3C and 3D). The attenuation of inflammatory response was also evident by decrease in levels of pro-inflammatory prompts, such as LTB_4_ (Figure 3E), cytokines and chemokine levels in the cortex (Figures 4A and 4B) and concomitant increase in the protective cytokine, IL-10 levels (Figure 4B). Interestingly, fenofibrate alone decreased the number of microglia (inset in Figure 3D), levels of LTB_4_ (inset in Figure 3E), cytokines and chemokine (insets in Figures 4A and 4B) suggesting the regulation of constitutive inflammmatory response by Cyp4fs, in a manner similar to that seen, in vitro, in BV-2 cells.

### Induction of Cyp4fs by fenofibrate attenuates JEV induced toxicity

**Figure 5:**
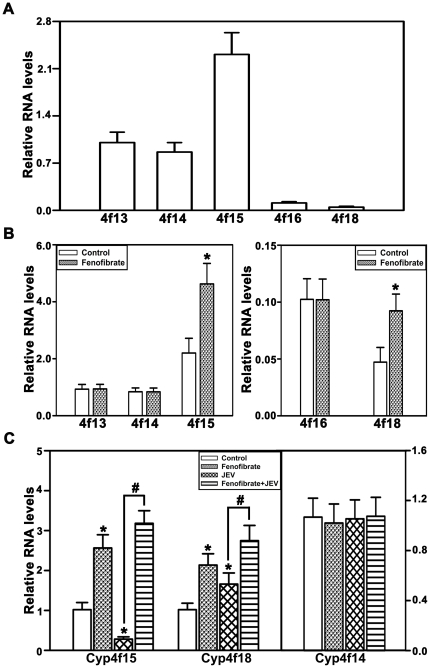
Effect of fenofibrate on Cyp4fs in mouse brain cortex with and without JEV challenge. (A) Constitutive expression of Cyp4fs in mouse brain cortex was assessed by qRT-PCR in BALB/c mice. Cyp4f15 was expressed maximally in mouse brain cortex. mRNA levels of all Cyp4fs are expressed relative to Cyp4f13 levels. (B) Treatment with fenofibrate significantly induced the expression of Cyp4f15 and 18 in mouse cortex, while Cyp4f13, 14 and 16 were unaltered. mRNA levels of all Cyp4fs are expressed relative to control Cyp4f13 levels. (C) JEV challenge significantly decreased Cyp4f15 but increased Cyp4f18 and Cyp4f14 was unaltered. Pretreatment with fenofibrate abolished JEV mediated decrease of Cyp4f15 and expression of both Cyp4f15 and 18 were enhanced. mRNA levels for all treatments are expressed relative to controls (n = 6 per group, *and #p<0.05).

In cortex of BALB/c mice, Cyp4f15 is constitutively the most abundantly expressed enzyme of the Cyp4f family, Cyp4f13 and Cyp4f14 are expressed at levels higher than Cyp4f16 and Cyp4f18 (Figure 5A). Cyp4f14, Cyp4f15 and Cyp4f18 are the most effective metabolizers of LTB_4_. In vivo, fenofibrate induced expression of Cyp4f15 and 18 while others remained unchanged (Figure 5B), unlike in BV-2 cells where all the Cyp4fs were induced with the exception of Cyp4f16. In mice, JEV infection alone significantly decreased the expression of Cyp4f15, which was abolished by pretreatment with fenofibrate. Pretreatment with fenofibrate further enhanced the increase in the levels of Cyp4f18 seen with JEV alone. Cyp4f14 levels were unaffected by JEV or fenofibrate (Figure 5C). It is likely that induction of Cyp4f15 and 18 by fenofibrate, which hydroxylate LTB_4_ efficiently could potentially contribute to the resolution of inflammation in the JEV infected mice (Figure 5C).

### Fenofibrate reduces viral replication

**Figure 6:**
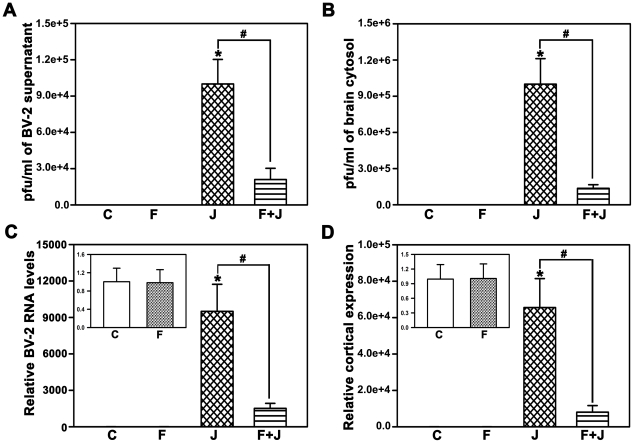
Fenofibrate reduces JEV replication both in vitro, in BV-2 cells and in vivo, in mouse brain. Infection with JEV significantly increased the viral titer in the culture medium of BV-2 cells (A) and the cytosol (B) prepared from the brains of BALB/c mice infected with JEV. Pretreatment with fenofibrate significantly decreased the JEV induced viral replication, both in vitro in BV-2 cells (A) and in vivo, in mouse brain (B). There was a corresponding decrease in the mRNA levels of GP78, the coat protein of JEV following fenofibrate treatment both in BV-2 cells (C) and in the brains of BALB/c mice (D) compared to JEV alone as quantitated by qRT-PCR. mRNA levels for all treatments are expressed relative to controls. Insets in C,D depict the levels of mRNA of GP78 in control and fenofibrate treated brains. Data is represented as (C) vehicle control, (F) fenofibrate, (J) JEV, (F+J) fenofibrate+JEV treated mouse brain cortex (n = 8 per group, *and #p<0.05).

BV-2 cells act as viral reservoirs supporting the replication and extrusion of JEV into the extracellular medium [26]. Pretreatment with fenofibrate for 4 days significantly decreased the viral titers in the culture medium from BV-2 cells infected with JEV (Figure 6A). mRNA levels of GP78, the viral coat protein also decreased significantly in the cells (Figure 6C, inset depicts the mRNA levels of GP78 between control and fenofibrate). In vivo, the viral titre was significantly lower in cytosol (Figure 6B) prepared from cortex of mice pretreated with fenofibrate for 4 days prior to JEV, as was the mRNA of GP78 (Figure 6D, inset depicts the mRNA levels of GP78 between control and fenofibrate).

### Pretreatment with fenofibrate for 2 days does not confer protection against JEV infection

**Figure 7:**
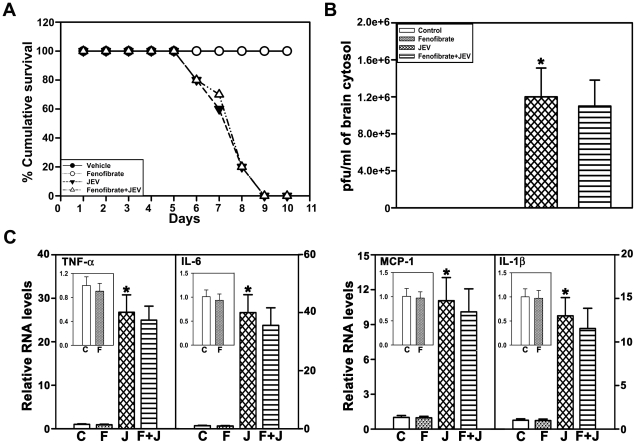
Pretreatment with fenofibrate for 2 days is not protective against JEV infection, in vivo, in mice. (A,B) Fenofibrate administered 2 days prior to JEV challenge has no effect on the mortality and viral titers in JEV infected animals. (C) mRNA expression of TNF-α, IL-6, MCP-1, IL-1β were unchanged in fenofibrate pretreated mice compared to those exposed to JEV alone. Insets in C show that cytokines and chemokine after fenofibrate treatment alone also were unchanged. Data is represented as (C) vehicle control, (F) fenofibrate, (J) JEV, (F+J) fenofibrate+JEV treated mouse brain cortex. mRNA levels in A,B for all treatments are expressed relative to controls (n = 8 per group, *and #p<0.05).

**Figure 8:**
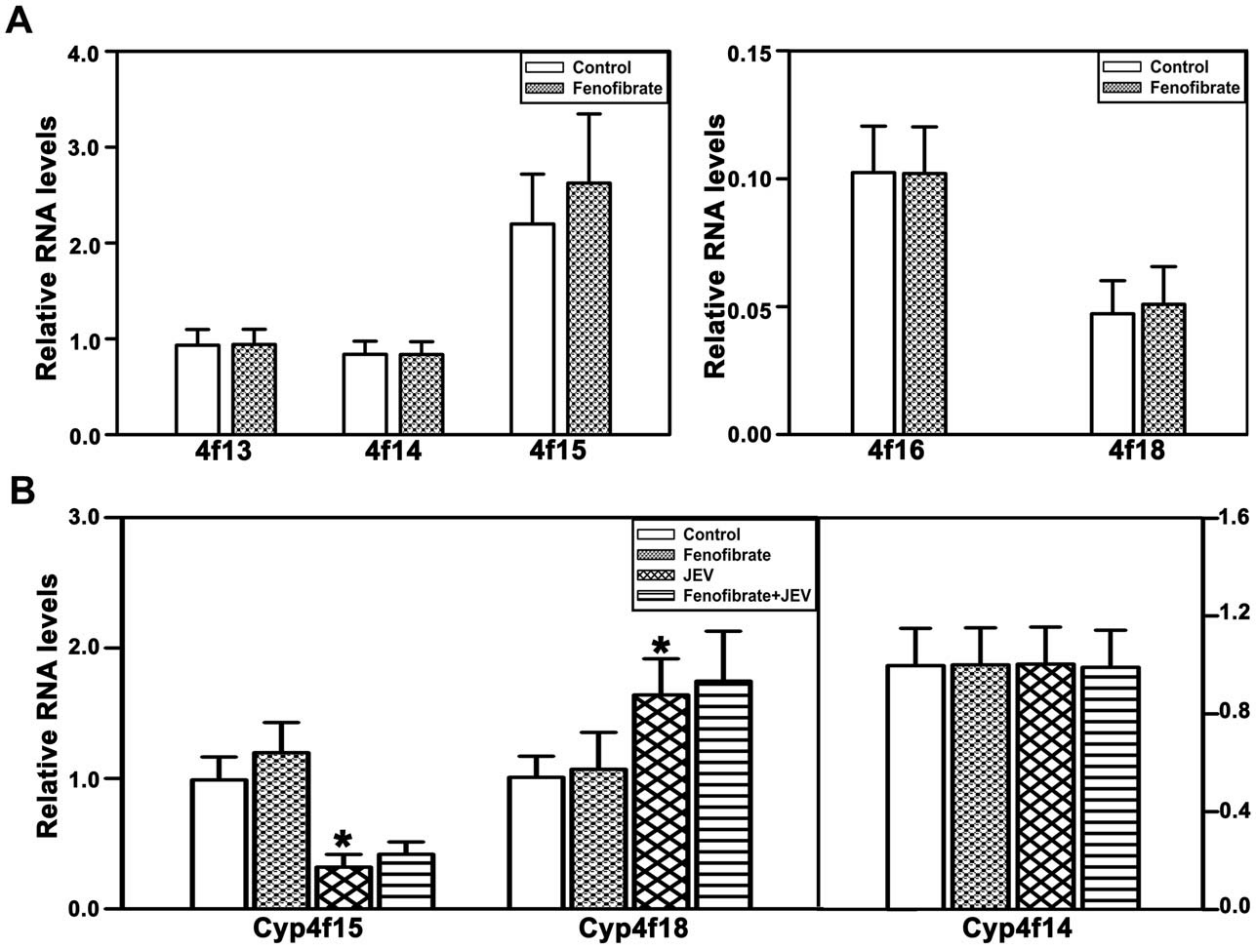
Cyp4fs were not induced following 2 days of fenofibrate pretreatment. (A) There was no change in the expression of Cyp4f13, 14, 15, 16 and 18 in mouse cortex following fenofibrate treatment for 2 days. mRNA levels of all Cyp4fs are expressed relative to control Cyp4f13 levels. (B) Pretreatment with fenofibrate did not enhance the levels of Cyp4f15 and 18 compared to mice exposed to JEV alone. mRNA levels for all treatments are expressed relative to controls (n = 8 per group, *and #p<0.05).

Prior exposure to fenofibrate for 2 days prior to JEV infection did not reduce mortality, the viral titers or the inflammatory response following JEV challenge (Figures 7A, 7B and 7C, respectively). Incidentally, pretreatment with fenofibrate for 2 days did not induce Cyp4fs and we did not find increase in Cyp4f15 and Cyp4f18 levels, which was evident following 4 days of pretreatment with fenofibrate (Figure 8A). Further, 2 days of fenofibrate exposure did not restore the substantial decrease observed in the expression of Cyp4f15 following JEV challenge (Figure 8B).

## Discussion

In the current study we demonstrate that fenofibrate, a well-known hypolipidemic drug when administered 4 days prior to JEV infection reduces mortality by 80% in a murine model of JEV infection and prevents neurological deficits in the surviving mice. Thus, fenofibrate could potentially be used as a prophylactic drug in endemic areas when clusters of JEV infections are seen in children, for example in the monsoon season.

Fenofibrate has been used in children for treating hyperlipidemia associated with Niemann-Pick disease, to improve insulin sensitivity and mitochondrial function following burn injury and as anti-angiogenic agent in children with brain tumors among others [27], [28], [29]. One of the side effects of the use of fenofibrate in adults is rhabdomyolosis, which is characterized by myalgia, weakness, dark urine and is a cause for acute renal failure [30], [31], [32], [33], [34]. However, while the use of fenofibrate in children has been described, there is no report of the occurrence of rhabdomyolosis [35]. Fenofibrate is known to cross BBB [18] and its administration 4 days prior to and 10 days post infection, which was necessary for the therapeutic effect did not cause any weight loss. In earlier studies also the neuroprotective effect of fenofibrate could be seen in apoE null mice only after 14 days of treatment prior to onset of cerebral ischemia [18].

PPARα is a member of ligand activated nuclear receptor family, which includes PPARβ/δ and PPARγ. Upon activation, they form heterodimer with retinoid X receptor (RXR) and activate transcription through peroxisome proliferator response elements (PPREs) in the promoter region of target genes [36], [37]. Fenofibrate is a well known PPARα agonist and mediates its action as a hypolipidemic agent by regulating the expression of a variety of modulators of lipid metabolism, such as apoA-I, apoA-II and lipoprotein lipase [38], [39], [40]. Fenofibrate also induces the expression of genes such as Cu/Zn^2+^ superoxide dismutase, glutathione peroxidase, glutathione reductase, glutathione-S-transferase and catalase in the brain and can thus, act as an effective anti-oxidant [20], [41], [42]. The mechanism underlying its anti-inflammatory effects, however are not clearly understood. It is known to interfere with NF-kB signaling by inducing expression of IκBα [43], [44]. In addition LTB_4_, the potent pro-inflammatory molecule that initiates, sustains and amplifies inflammation has been shown to be a ligand for PPARα. LTB_4_ through its action as PPARα agonist can enhance its metabolism, thus diminishing the inflammatory response [45]. However, when the production of LTB_4_ is overwhelming then more potent agonist of PPARα may help enhance the metabolism of LTB_4_ thus reducing the inflammatory response.

Cytochrome P450 (Cyp), a superfamily of heme proteins is involved in the metabolism of xenobiotics and endogenous compounds. Cytochrome P4504F (Cyp4f) subfamily includes 7 functional enzymes in humans, 4 in rat and 5 in mice [46], [47]. Recombinant Cyp4f14, Cyp4f15 and Cyp4f18 are the most effective members of Cyp4f sub-family for LTB_4_ metabolism, while Cyp4f13 and 16 are poor metabolizers [21]. Recently, we demonstrated that the neuroprotective action of fenofibrate in a murine model of LPS mediated neuro-inflammation occurred through its ability to induce selective Cyp4fs that effectively hydroxylated LTB_4_ [21]. Therefore, we examined the levels of Cypfs, particularly Cyp4f14, 15 and 18 in the murine model of JEV infection. Cyp4f15 and 4f18 are induced by fenofibrate while 4f13, 14 and 16 are unchanged (Figure 5B). Surprisingly, following JEV infection, Cyp4f15 is significantly down-regulated whereas 4f18 levels are increased in mouse brain (Figure 5C). However, prior administration of fenofibrate enhances the levels of 4f15 and 18, thus potentially increasing the metabolism of LTB_4_ to 20-hydroxy LTB_4_ and a corresponding decrease in the levels of cytokines and chemokine. Interestingly we found that JEV infection per se decreases the levels of Cyp4f15 in a manner quite distinctive from the effects of LPS wherein 4f15 was induced [21]. The mechanism underlying this distinctive effect of JEV infection is yet to be understood.

Fenofibrate is known to inhibit the replication of Herpes simplex virus Type 1 and human immunodeficiency virus. The antiviral activity of fenofibrate is associated with the activation of PPARα receptors [48], [49]. Therefore, we examined the potential of fenofibrate to inhibit JEV replication and found a profound reduction in the viral titers both in vitro and in vivo when fenofibrate was administered 4 days prior to JEV challenge (Figure 6). To elucidate its mode of action, fenofibrate was also administered 2 days prior to JEV infection and found that it had no effect on the mortality, inflammatory response or viral replication quite unlike the dramatic protective effects seen when it was administered 4 days prior to JEV infection. Interestingly, this treatment paradigm also did not have any inducing effect on the Cyp4fs unlike the 4 days treatment with fenofibrate. Thus, the induction of Cyp4f15 observed after 4 days of fenofibrate pretreatment correlates with the neuroprotective effect seen, while shorter pretreatment which has no effect on Cyp4fs does not have any effect on the neurotoxicity seen after JEV infection. Therefore, it seems plausible that induction of critical genes including Cyp4fs through activation of PPARα is required for the anti-viral and anti-inflammatory effects seen following fenofibrate treatment. Nevertheless, the substantial anti-viral effect of fenofibrate demonstrated in this study is of significance and points to its potential therapeutic role in controlling JEV infection.

One might question the usefulness of prophylaxis of drug if vaccination against JEV is possible. The inactivated mouse-brain derived, inactivated cell culture derived and live attenuated cell-culture derived are the three types of JE vaccines used for immunization. However, their use is limited in terms of availability, cost and safety [50], [51], [52], [53], [54], [55]. In view of this, use of prophylatic drugs with good safety profile may be more effective in dealing with epidemic-like situation in endemic areas.

In conclusion, we hereby demonstrate the effectiveness of fenofibrate administered prior to JEV infection to reduce mortality and prevent neurological dysfunction in survivors in murine model of JE. Given the prevalence of this infection in endemic areas, the high mortality and morbidity that it causes among children and the lack of effective curative therapies, fenofibrate with its well established safety profile could offer an inexpensive, safe and effective prophylactic therapy for JEV infection.
